# Bioactivity screening of plant ethanol extracts against *Plutella xylostella* and field evaluation of an optimized botanical combination formulation

**DOI:** 10.3389/abp.2026.16918

**Published:** 2026-08-27

**Authors:** Jie Zhang, Xiaoe Wang, Xiaoli Xu, Williamson Gustave, Xiaokai Zhang

**Affiliations:** 1 College of Yizhi Agriculture and Forestry, Xianyang Polytechnic Institute, Xianyang, Shaanxi, China; 2 Zhejiang Ecopro Agro Technology Co., Ltd., Jiaxing, Zhejiang, China; 3 School of Chemistry, Environmental and Life Sciences, University of the Bahamas, Nassau, Bahamas; 4 Institute of Environmental Processes and Pollution Control, School of Environment and Ecology, Jiangnan University, Wuxi, China

**Keywords:** antifeedant activity, botanical insecticide, ethanol extract, field control efficacy, Plutella xylostella

## Abstract

*Plutella xylostella* is a major pest of cruciferous vegetables. The prolonged overuse of chemical insecticides has resulted in increased resistance in *P. xylostella* populations, highlighting the urgent need for environmentally friendly and effective alternative control strategies. In this study, ethanol extracts from 15 plant species collected from the Qinling Mountains of China were systematically evaluated for their insecticidal, antifeedant, and oviposition-repellent activities against *P. xylostella.* Laboratory bioassays were conducted using leaf-dipping and leaf-disc methods. Among the tested plants, ethanol extracts of *Atropa belladonna* and *Blumea riparia var. megacephala* exhibited the strongest biological activities. Specifically, *A. belladonna* extract achieved corrected mortality, antifeedant, and oviposition-repellent rates of 83.11%, 89.46%, and 78.57%, respectively, whereas *B. riparia var. megacephala* extract produced corrected mortality, antifeedant, and oviposition-repellent rates of 73.53%, 87.45%, and 82.14%, respectively. When combined at a volume ratio of 8:2, the two extracts showed enhanced insecticidal activity, with a median lethal concentration (LC50) of 65.762 mg/mL and a co-toxicity coefficient consistent with an additive effect without antagonism. A stable emulsifiable concentrate (EC) formulation was subsequently developed through optimization of the solvent and surfactant system. Field trials demonstrated that the 10% botanical EC effectively suppressed *P. xylostella* populations, achieving a control efficacy of 58.35% at 7 days post-application, which was comparable to that of the commonly used insecticide emamectin benzoate. Collectively, these findings suggest that *A. belladonna* and *B. riparia var. megacephala* have potential as botanical insecticide resources for the integrated management of *P. xylostella* and may contribute to more sustainable agricultural practices.

## Introduction

*Plutella xylostella* is characterized by strong environmental adaptability and high reproductive capacity. Adults are small-bodied insects with well-developed wing venation and dense scales and are capable of long-distance migration, making *P. xylostella* one of the most widely distributed and destructive lepidopteran pests worldwide (Yang et al., 2024). The life cycle of *P. xylostella* comprises four stages: egg, larva, pupa, and adult. Outbreaks primarily occur during spring and autumn, and the larvae feed mainly on cruciferous vegetables (Capinera, 2008). After mating, female adults lay eggs on the underside of leaves. Upon hatching, the larvae initially feed on the abaxial leaf surface. From the second instar onward, larvae either continue feeding on the leaf underside or bore into internal plant tissues. Feeding activity becomes particularly intense during the third and fourth instars and may result in severe defoliation (Bhure et al., 2020). Due to its high reproductive rate and overlapping generations, *P. xylostella* can damage crops throughout the entire growing season in cruciferous production regions, leading to substantial reductions in crop yield and quality and, in severe cases, complete crop loss (Oplopoiou et al., 2024). In China, the prolonged of chemical insecticides has imposed strong selective pressure on *P. xylostella* populations, resulting in widespread insecticide resistance. Consequently, *P. xylostella* is now regarded as one of the most insecticide-resistant agricultural pests worldwide.

Since Ankersmit first reported resistance of *P. xylostella* to dichlorodiphenyltrichloroethane (DDT) in 1950, this pest has developed varying degrees of resistance to nearly 100 insecticides, including organophosphates, amidinourea derivatives, and oxadiazines (Ankersmi, 1953; Roditakis et al., 2017). Available evidence indicates that resistance has been reported for more than 90% of the insecticides used against Chinese *P. xylostella* populations, often accompanied by cross-resistance. For example, *P. xylostella* populations in the Yangtze River Delta region exhibit moderate to high resistance to multiple insecticides (Sun et al., 1978). Populations in Jiangxi Province have developed high resistance to abamectin and related compounds (Iqbal et al., 1996), whereas populations in Gansu Province show resistance to both abamectin and cyfluthrin (Jin et al., 2021). Furthermore, *P. xylostella* populations in Baiyun District, Guangdong Province, display high resistance to indoxacarb and moderate resistance to metaflumizone (Wang et al., 2016). Previous studies have confirmed cross-resistance in *P. xylostella* populations; for instance, strains resistant to chlorantraniliprole exhibit moderate resistance to cyantraniliprole (Zhang et al., 2014; Zhang, 2016).

In response to the growing threat of insecticide resistance in *P. xylostella*, increasing attention has been directed toward the development of plant-derived insecticidal compounds as alternative control strategies. Many plants can produce secondary metabolites that interfere with insect growth, development, reproduction, and physiological functions, and these bioactive components form the basis of many botanical insecticides (Benelli and Maggi, 2022). For example, plant-derived alkaloids, terpenoids, and phenolic compounds exhibit insecticidal activity through multiple mechanisms, including disruption of neural transmission, inhibition of feeding and oviposition behavior, and interference with insect metabolic processes (Divekar et al., 2022). Alkaloids primarily act on neural receptors and ion channels, terpenoids are known for their antifeedant and repellent properties, and phenolic compounds may inhibit insect growth and development through oxidative or toxic effects (Farhan et al., 2024). In addition, botanical insecticides have been considered promising alternatives because of their biodegradability, diverse bioactive constituents, and potential value in resistance-management strategies when used appropriately in integrated pest management programs (Dolma et al., 2021; Pengsook et al., 2022). Consequently, botanical insecticides have emerged as a promising approach for the sustainable management of *P. xylostella* in vegetable production systems.

To explore alternative strategies for managing *P. xylostella*, this study investigated the insecticidal potential of ethanol extracts from 15 plant species collected in the Qinling Mountains of China. Extracts exhibiting high biological activity during preliminary screening were further evaluated through laboratory bioassays and field trials to assess their control efficacy. The aim of this study was to identify potential botanical insecticide resources and to explore environmentally sustainable management strategies for *P. xylostella*.

## Materials and methods

### Collection of plant materials and preparation of extracts

Fifteen plant species were selected from the Qinling Mountains of China based on local resource reserves and representative regional distribution. The selection was intended to explore native plant resources with potential pesticidal activity rather than to provide an exhaustive survey of all insecticidal plants in the region. The selected species were: *Solanum nigrum* L., *Atropa belladonna* L., *Fritillaria cirrhosa* D. Don, *Allium anisopodium* Ledeb., *Asparagus dauricus* Link, *Allium ochotense* Prokh., *Tupistra wattii* Hook. f., *Eupatorium japonicum* Thunb, *Aucklandia lappa* Decne., *Blumea riparia* var. *megacephala* Randeria, *Ligularia dolichobotrys* Diels, *Bauhinia championi* Benth., *Cassia tora* L., *Abrus precatorius* L., *Lespedeza davurica* (Laxm.) Schindl. Collected plant materials were rinsed with tap water to remove soil and debris, and diseased or damaged tissues were discarded. The materials were air-dried in a well-ventilated area until completely dry and then further dried in a constant-temperature oven at 45 °C. The dried materials were ground into powder, passed through a 0.355 mm sieve, sealed, and stored at 25 °C until use.

At room temperature, 50 g of dried plant powder was weighed and mixed with 95% ethanol at a solid-to-liquid ratio of 1:4 (w/v). The mixture was extracted for 48 h with periodic stirring. After filtration, the residue was re-extracted twice under the same conditions using fresh ethanol. The combined extracts were filtered through a Büchner funnel, and the residue was rinsed two to three times until the filtrate became clear. The filtrate was concentrated using a rotary evaporator (210 rpm, water bath at 35 °C) to obtain the crude extract, which was subsequently weighed, sealed, and stored under refrigeration (Khasanah et al., 2021). For experimental use, the crude extract was dissolved in ethanol to prepare a 200 mg/mL stock solution and then diluted to concentrations of 25, 50, 75, 100, and 150 mg/mL.

### Rearing of test insects

Healthy third-instar larvae of *P. xylostella* of similar size were selected for laboratory bioassays. All larvae were maintained in a climate-controlled chamber at 25 °C ± 1 °C, 60% ± 10% relative humidity, and a photoperiod of 14 h light: 10 h dark (14L: 10D). To minimize variation in feeding status and ensure experimental consistency, larvae were starved for 4 h prior to each bioassay to standardize feeding conditions (Zheng et al., 2023).

### Toxicity and behavioral bioassays of test insects

#### Toxicity bioassay

The insecticidal activity of ethanol extracts from 15 plant species against *P. xylostella* was preliminarily screened at 50 mg/mL using the leaf-dipping method, with this concentration determined based on existing studies (Ibrahim et al., 2022; Zheng et al., 2023). Cabbage leaves were cut into circular leaf discs (5 cm in diameter), immersed in the test solutions for 10 s, and air-dried at room temperature. The treated leaf discs were placed into glass Petri dishes lined with moistened filter paper. Leaf discs treated with ethanol alone served as the control (CK). Twenty healthy third-instar *P. xylostella* larvae, starved for 4 h prior to the assay, were introduced into each dish. Each treatment was performed with three independent biological replicates. The replicate dish, rather than each individual larva, was treated as the experimental unit for statistical analysis. Larval mortality was recorded at 24, 48, and 72 h after treatment, and corrected mortality was calculated using Equation 1.

$$CM=\frac{M_{T}-M_{C}}{1-M_{C}}\times 100\%$$ (1)

Where *CM* represents the corrected mortality rate (%), *M*_*C*_ represents the mortality rate of the control group (%), and *M*_*T*_ represents the mortality rate of the treatment group (%).

#### Antifeedant activity assay

The antifeedant activity of ethanol extracts from different plant species against *P. xylostella* larvae was evaluated using the leaf-disc method (Singh et al., 2023). Cabbage leaves were cut into uniform leaf discs (1.5 cm in diameter), immersed into the test solutions (50 mg/mL) for 10 s, air-dried, and then placed into Petri dishes (12 cm in diameter) lined with moistened filter paper. Leaf discs treated with ethanol alone served as the CK. Four treated leaf discs were arranged alternately in each dish, and two healthy third-instar larvae, starved for 4 h prior to the assay, were introduced into each dish. Each treatment was performed with five independent biological replicates, and each replicate dish, rather than individual larvae, was treated as the experimental unit for statistical analysis. After 48 h of exposure, the leaf area consumed by the larvae was measured, and the antifeedant rate was calculated using Equation 2.

$$AR=\frac{A_{C}-A_{T}}{A_{C}}\times 100\%$$ (2)

where *AR* represents the antifeedant rate (%), *A*_*C*_ represents the leaf area consumed in the control group (mm^2^), and *A*_*T*_ represents the leaf area consumed in the treatment group (mm^2^).

#### Oviposition repellency assay

Pot-grown Chinese cabbage plants at the 4–5 true-leaf stage and exhibiting uniform growth were selected for the assay. Each plant was sprayed with 5 mL of plant ethanol extract (50 mg/mL) until the leaf surfaces were thoroughly wetted and droplets formed. After treatment, the plants were air-dried at room temperature. Plants sprayed with an equal volume of ethanol served as the CK. The treated plants were individually placed in insect-rearing cages (100 cm × 80 cm × 80 cm), and 20 adult *P. xylostella* moths were introduced into each cage (Cerda et al., 2019). A 0.5% honey solution was provided as a food source for the adults. Each treatment consisted of four independent biological replicates, and the rearing cage was used as the experimental unit for statistical analysis. After 24 h of exposure, the number of eggs laid on each cabbage plant was recorded, and the oviposition repellency rate was calculated using Equation 3.

$$ODR=\frac{F_{C}-F_{T}}{F_{C}}\times 100\%$$ (3)

where *ODR* represents the oviposition repellent rate (%), *F*_*C*_ represents the number of eggs laid in the control group, and *F*_*T*_ represents the number of eggs laid in the treatment group.

#### Toxicity bioassay of combined plant extracts

Based on the toxicity and behavioral bioassays described above, *A. belladonna* and *B. riparia var. megacephala* were selected for combination toxicity screening, as these two extracts exhibited the most prominent overall activity among the 15 tested plant extracts in terms of larvicidal toxicity, antifeedant activity, and oviposition repellency against *P. xylostella*. To maintain a clear and interpretable combination system, a binary mixture design was adopted for evaluating the interaction between the two most active extracts. In total, eleven extract treatments were prepared in a volumetric series, including two single-extract treatments and nine binary mixtures. The volume ratios of *A. belladonna* extract to *B. riparia var. megacephala* extract were 10:0, 9:1, 8:2, 7:3, 6:4, 5:5, 4:6, 3:7, 2:8, 1:9, and 0:10 (v/v). The toxicity of each mixture against *P. xylostella* larvae was evaluated using the method described in Section *Toxicity bioassay*. According to the Chinese agricultural industry standard Pesticide Indoor Bioassay Test Guidelines (NY/T 1154.7-2006, 2026), the co-toxicity coefficient (CTC) of each mixture was calculated using Equation 4. CTC values were interpreted as follows: CTC ≥120 indicated synergism, CTC ≤80 indicated antagonism, and CTC values between 80 and 120 indicated an additive effect (Zhang et al., 2022).

$$CTC=\frac{\left(S/M\right)\times 100}{TI_{A}\times P_{A}+TI_{B}\times P_{B}}$$ (4)

Where *CTC* represents the co-toxicity coefficient; *S* represents the median lethal concentration (LC_50_) of the reference insecticide; *M* represents the median lethal concentration (LC_50_) of the combined mixture; *TI*_*A*_ is the toxicity index of compound A; *P*_*A*_ represents the volume proportion of compound A in the mixture; *TI*_*B*_ represents the toxicity index of compound B; and *P*_*B*_ represents the volume proportion of compound B in the mixture.

### Formulation development and stability testing

A botanical emulsifiable concentrate (EC) formulation was developed using extracts of *A. belladonna* and *B. riparia var. megacephala* as the active ingredients. Four organic solvents, dimethyl sulfoxide, methanol, ethanol, and petroleum ether, were evaluated for their ability to dissolve the plant extracts. In addition, four surfactants (pesticide emulsifiers 500#, 600#, and 601#, and OP-10) were tested to assess their emulsification performance.

The two plant extracts were combined at a volume ratio of 8:2. The mixed extract was added to test tubes containing different organic solvents for solubility screening. After standing at room temperature for 4 h, each solution was examined for instability, including precipitation or phase separation, to identify solvents capable of maintaining a clear and homogeneous system. Suitable solvents were subsequently selected for formulation preparation. After complete dissolution of the combined extracts, the solutions were mixed with different surfactant combinations to prepare EC formulations. The formulations were allowed to stand at room temperature for 24 h and were then subjected to high- and low-temperature stability tests to evaluate emulsification stability. Surfactant combinations that maintained homogeneous, transparent, and physically stable systems under both temperature conditions were selected for the final EC formulation.

For the high-temperature stability test, 30 mL of the EC sample was transferred into a sterile ampoule. The ampoule was precooled in an ice-salt bath, flame-sealed to prevent solvent evaporation, and stored at 54 °C ± 2 °C for 14 days. After storage, the formulation was examined for abnormalities such as phase separation, precipitation, or deterioration to assess thermal stability (GB/T 19136-2021, 2026). For the low-temperature stability test, 100 mL of the EC sample was transferred into a centrifuge tube and gradually cooled to 1 °C. During the first hour, the sample was stirred for 20 s every 15 min while monitoring for precipitation. The sample was then stored at 0 °C ± 1 °C for 7 days. After returning to room temperature over 3.5 h, the sample was centrifuged for 15 min, and the precipitate volume was measured to a precision of 0.03 mL. A precipitate volume of less than 0.30 mL was considered acceptable for low-temperature stability of the EC formulation (GB/T 19137-2003, 2026).

### Field experimental design

Field efficacy trials were conducted in accordance with the Chinese agricultural industry standard guidelines for field efficacy trials of pesticides (GB/T 17980.1-2000, 2026). The experiments were performed at an experimental site in Jingyang County using the cruciferous vegetable cultivar Qinggeng Songhua 65. Uniform cultivation and field management practices were maintained throughout the experiment. No detectable insecticide resistance was observed in the local *P. xylostella* population. Experimental plots were separated from surrounding areas by wasteland buffer zones, and applications were performed under overcast weather conditions. Three treatments were evaluated: Treatment 1, a 10% *A. belladonna-B. riparia var. megacephala* EC diluted 100-fold before application; Treatment 2, a 1% emamectin benzoate microemulsion diluted 1000-fold and used as the positive control; and Treatment 3, water spray used as the CK. Each treatment was replicated four times, resulting in a total of 12 plots, each measuring 20 m^2^. The experiment was arranged in a randomized complete block design. A five-point sampling method was used, with five sampling points established within each plot and two fixed plants assessed at each sampling point. The number of surviving *P. xylostella* larvae was recorded before treatment and at 1, 3, and 7 days after application. Larval population reduction rate and field control efficacy were calculated using Equations 5, 6, respectively.

$$DR=\frac{N_{0}-N_{t}}{N_{0}}\times 100\%$$ (5)

Where *DR* represents the population reduction rate (%), *N*_*0*_ is the number of living larvae before treatment (individuals per plot), and *N*_*t*_ is the number of living larvae on day t after treatment (individuals per plot).

$$E=\frac{DR_{T}-DR_{C}}{1-DR_{C}}\times 100\%$$ (6)

Where *E* represents the control efficacy (%), *DR*_*T*_ denotes the population reduction rate in the treatment group (%), and *DR*_*C*_ denotes the population reduction rate in the control group (%).

### Statistical analysis

All experiments were conducted with at least three independent biological replicates for each treatment, as specified in the corresponding assay sections. Data are presented as mean ± standard deviation (SD). Statistical analyses were performed using SPSS Statistics 27 software (IBM, USA). Differences among multiple treatments were evaluated using Duncan’s multiple range test, while independent-samples t-tests were used for pairwise comparisons where appropriate. Statistical significance was defined at *P* < 0.05. Figures were generated using Origin 2024 software (OriginLab, USA).

## Results

### Toxicity of ethanol extracts from different plants against *P. xylostella* larvae

**FIGURE 1:**
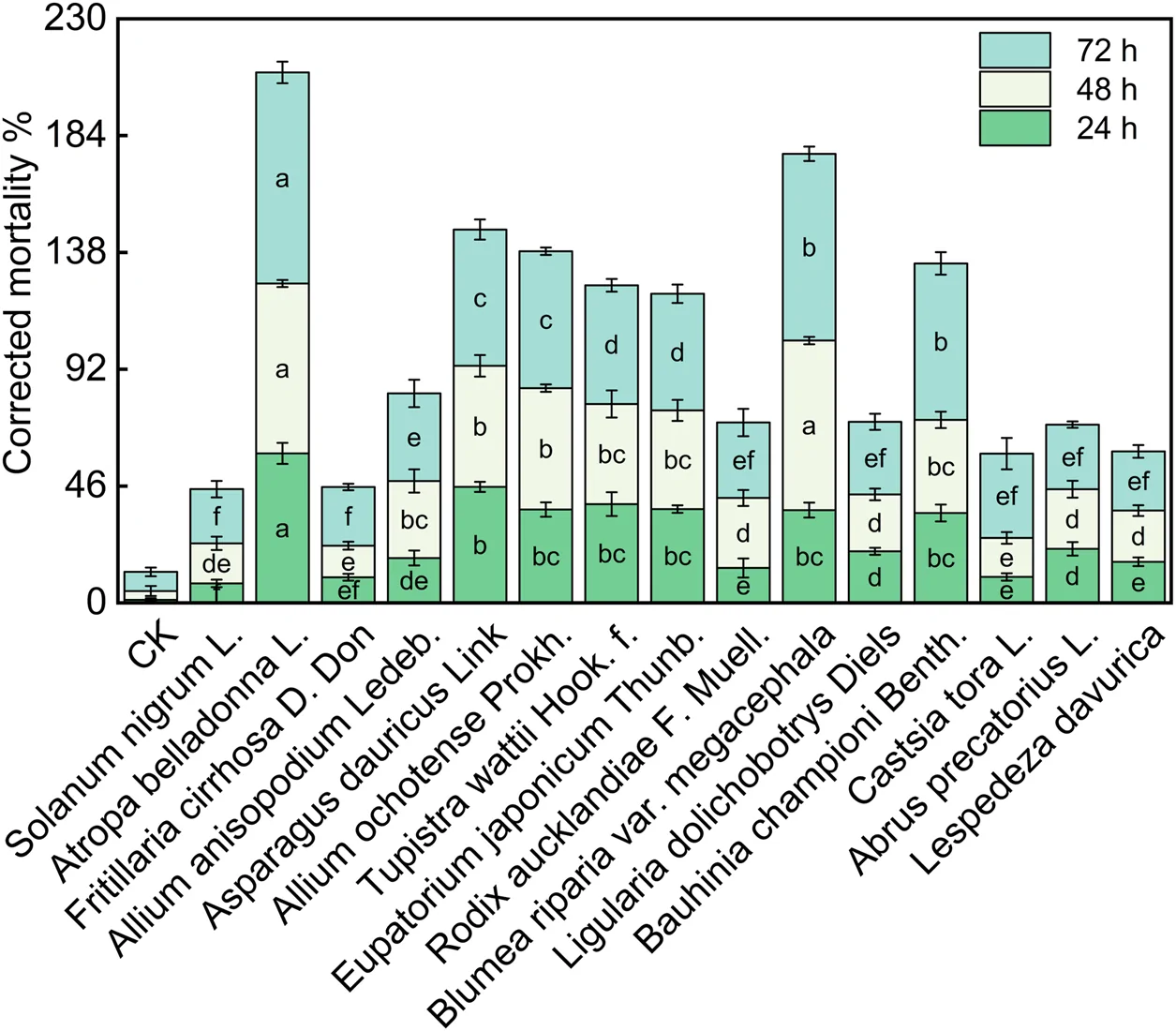
Insecticidal activity of ethanol extracts from different plant species (50 mg/mL) against *P. xylostella* larvae. Different letters indicate significant differences (one-way ANOVA, Duncan’s test, *p* < 0.05).

In this study, the insecticidal activity of ethanol extracts from 15 plant species against *P. xylostella* larvae was preliminarily screened at 50 mg/mL using the leaf-dipping method (Figure 1). Significant interspecific differences in insecticidal activity were observed among the tested plant extracts, and several highly active extracts exhibited a clear time-dependent effect. At 24 h post-treatment, the *A. belladonna* extract produced a corrected mortality rate of 58.87%, which was significantly higher than that of all other tested extracts (*P* < 0.05), indicating that it was the most effective treatment at this time point. The extracts of *A*. *dauricus* (45.67%), *A*. *ochotense* (36.77%), *T*. *wattii* (38.89%), and *B. riparia* var. *megacephala* (36.55%) also exhibited corrected mortality rates above 35%. In contrast, the remaining ten plant extracts showed corrected mortality rates below 30%, with *S*. *nigrum* exhibiting the lowest activity (7.69%), which was not significantly different from CK (*P* > 0.05).

After 48 h of treatment, the insecticidal activity of the *A. belladonna* extract further increased, reaching a corrected mortality rate of 66.89%, while the corrected mortality rate of *B. riparia* var. *megacephala* increased to 66.74%. No significant difference was observed between these two extracts (*P* > 0.05), and both exhibited significantly greater insecticidal activity than the other treatment groups (*P* < 0.05). The corrected mortality rates of *A*. *dauricus*, *A*. *ochotense*, *T*. *wattii*, and *E*. *japonicum* extracts ranged from 38.86% to 47.79%, indicating moderate insecticidal activity, with only slight increases compared to the 24 h time point. In contrast, the corrected mortality rates of the remaining plant extracts remained below 30%, indicating relatively weak insecticidal activity under the conditions tested.

After 72 h of treatment, the insecticidal activity of the *A. belladonna* extract further increased, reaching a corrected mortality rate of 83.11%. This was the only extract to achieve a corrected mortality rate exceeding 80% and was significantly higher than all other treatments (*P* < 0.05). The corrected mortality rate of *B. riparia* var. *megacephala* also increased to 73.53%, representing the second-highest activity among the tested extracts. The extracts of *B*. *championi*, *A*. *dauricus*, and *A*. *ochotense* exhibited corrected mortality rates of 61.59%, 53.66%, and 53.91%, respectively, indicating moderate to high insecticidal activity. In contrast, the remaining ten plant extracts produced corrected mortality rates below 50%, indicating relatively limited insecticidal activity under the conditions tested. Overall, the ethanol extracts of *A. belladonna* and *B. riparia* var. *megacephala* demonstrated the strongest and most consistent insecticidal activity against third-instar *P. xylostella* larvae, with mortality increasing over time.

### Antifeedant activity of ethanol extracts from different plants against *P. xylostella* larvae

**FIGURE 2:**
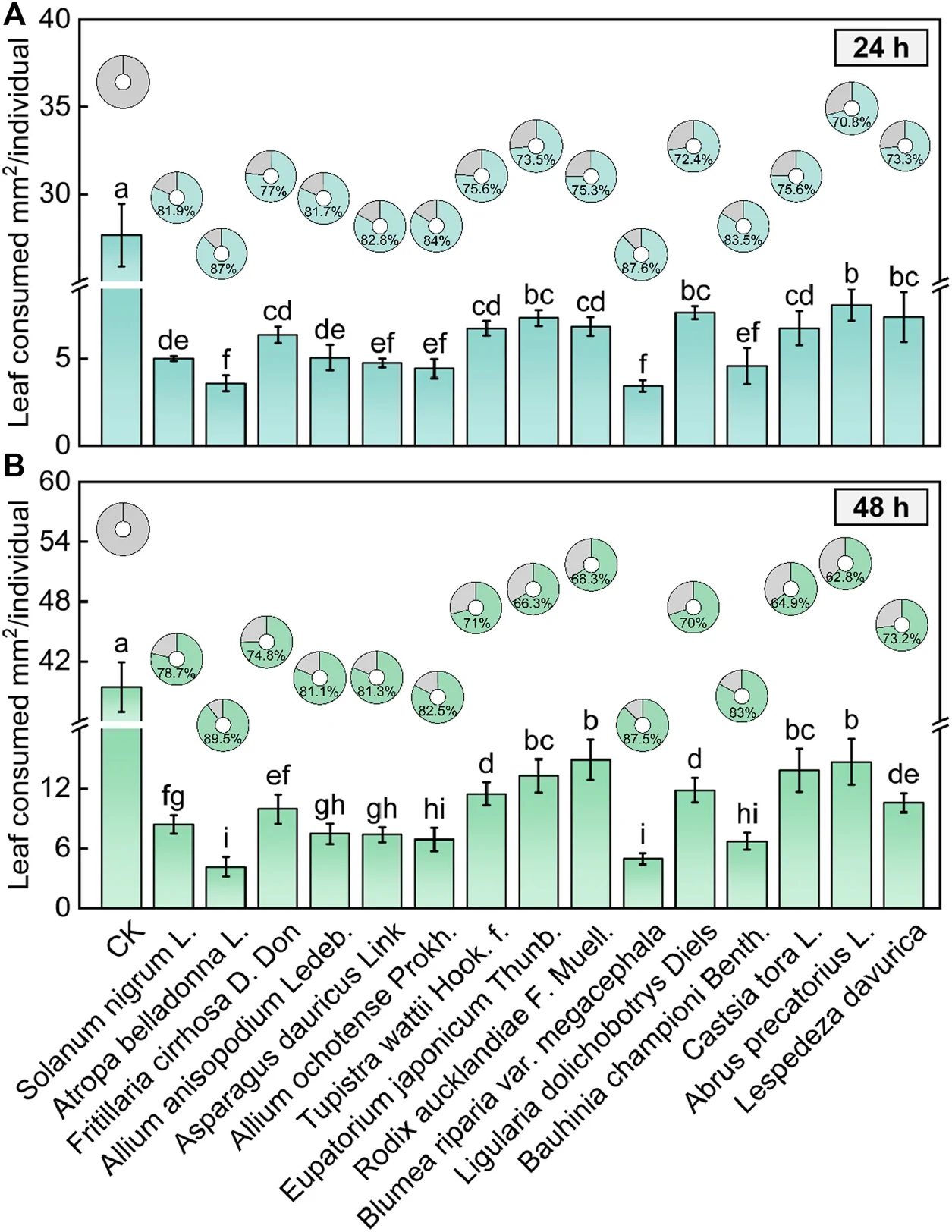
Antifeedant effects of ethanol extracts from different plant species (50 mg/mL) against *P. xylostella* larvae. Bar graphs **(A,B)** show the leaf consumption by larvae under the influence of different plant extracts at 24 h and 48 h, respectively; the pie charts represent the larval antifeedant rates. Different letters indicate significant differences (one-way ANOVA, Duncan’s test, *p* < 0.05).

As shown in Figure 2, significant differences in antifeedant activity against *P. xylostella* larvae were observed among the ethanol extracts from the 15 plant species. The results at 24 h and 48 h demonstrated that the extracts of *B. riparia* var. *megacephala* and *A. belladonna* possessed the strongest and most stable antifeedant effects. At 24 h, larval leaf consumption in the *B. riparia* var. *megacephala* and *A. belladonna* treatments was 3.44 mm^2^/larva and 3.58 mm^2^/larva, respectively, corresponding to antifeedant rates of 87.55% and 87.04%. At 48 h, leaf consumption remained low at 4.95 mm^2^/larva and 4.16 mm^2^/larva, while antifeedant rates reached 87.45% and 89.46%, respectively. Both extracts exhibited significantly greater antifeedant activity than the other treatment groups (*P* < 0.05). These findings are generally consistent with the insecticidal activity results described above.

The extracts of *A*. *ochotense*, *A*. *dauricus*, and *B*. *championi* also exhibited strong antifeedant activity, with antifeedant rates of 83.97%, 82.81%, and 83.46% at 24 h, respectively. Although these values declined slightly over time, they remained above 80% at 48 h. In contrast, the extracts of *A*. *precatorius* and *L*. *dolichobotrys* exhibited the weakest antifeedant activity, with antifeedant rates of only 70.80% and 72.39% at 24 h. Additionally, the antifeedant activity of *A*. *lappa* and *C*. *tora* extracts declined substantially over time, with antifeedant rates dropping to 62.26% and 64.92% at 48 h. Taken together, the extracts of *A. belladonna* and *B. riparia* var. *megacephala* exhibited potent antifeedant activity against *P. xylostella* larvae.

### Oviposition repellent activity of different plant ethanol extracts against *P. xylostella*

**FIGURE 3:**
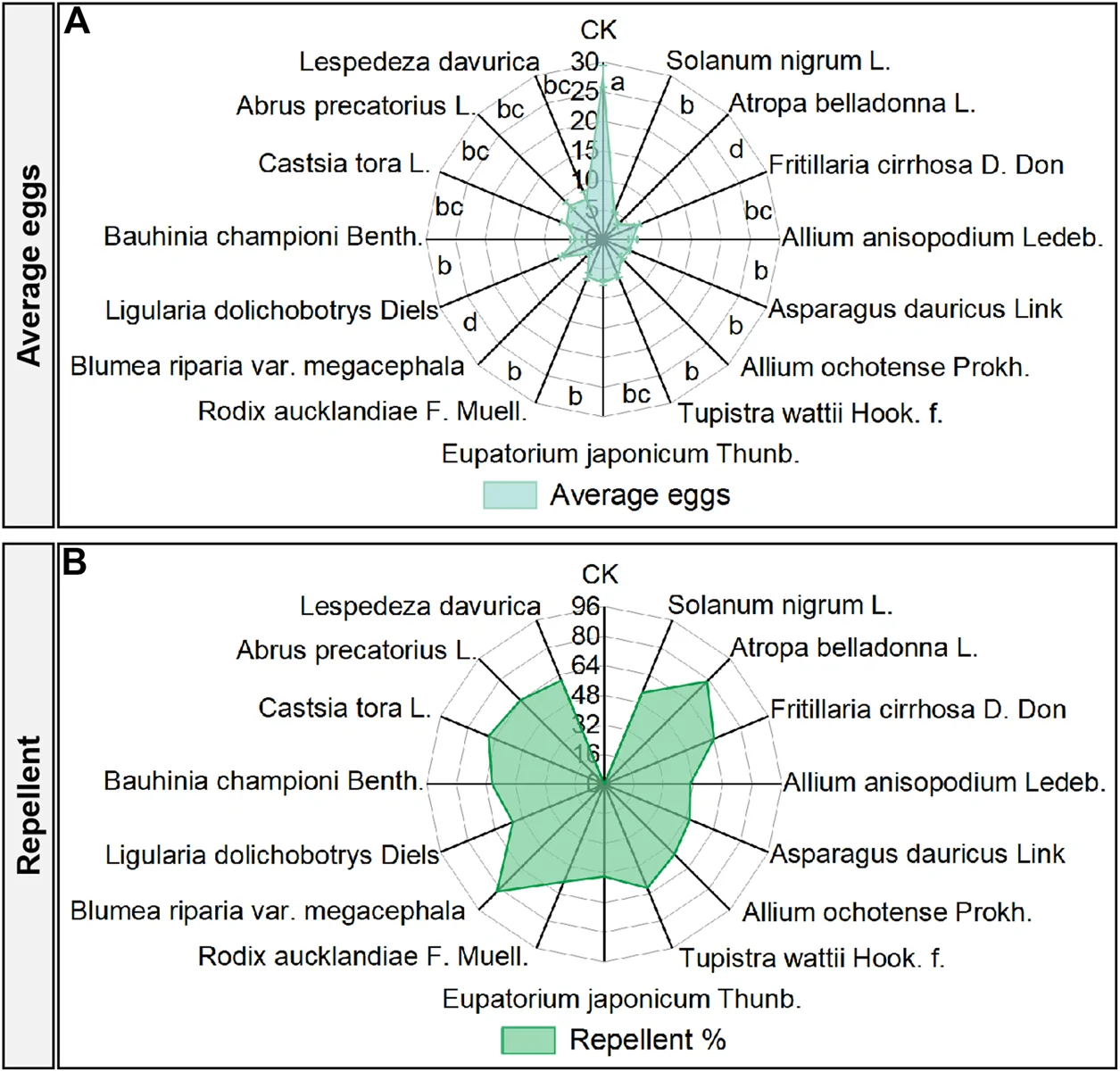
Oviposition-repellent activity of ethanol extracts from different plant species (50 mg/mL) against *P. xylostella*. **(A,B)** show the average number of eggs laid and the oviposition repellency rate, respectively. Different letters indicate significant differences (one-way ANOVA, Duncan’s test, *p* < 0.05).

As shown in Figure 3, significant differences in oviposition repellent activity against *P. xylostella* were observed among the tested plant extracts. The extracts of *B. riparia* var. *megacephala* and *A. belladonna* exhibited the most prominent repellent effects, consistent with their previously demonstrated larvicidal and antifeedant activities. On the CK cabbage plants, *P. xylostella* adults laid an average of 7.00 eggs per plant, providing a stable baseline for normal oviposition behavior. All plant extract treatments reduced oviposition by *P. xylostella* to varying degrees.

Among the tested extracts, the *B. riparia* var. *megacephala* extract produced the lowest oviposition count, at only 1.25 eggs per plant, corresponding to an oviposition repellent rate of 82.14%. The *A. belladonna* extract showed the second-highest repellent effect, with 1.50 eggs per plant and a repellent rate of 78.57%. Both were significantly superior to all other treatments (*P* < 0.05). The extracts of *C*. *tora*, *F*. *cirrhosa*, *A*. *precatorius*, *T*. *wattii*, *B*. *championi*, and *L*. *davurica* exhibited moderate oviposition repellent activity, with repellent rates of 60.71%–67.86% and oviposition counts below 3.00 eggs per plant. In contrast, the extracts of *A*. *ochotense*, *A*. *dauricus*, *E*. *japonicum*, *A*. *lappa*, *S*. *nigrum*, and *A*. *anisopodium* showed weaker repellent activity, with oviposition repellent rates of 46.43%–57.14%. Among these, *A*. *anisopodium* had the weakest repellent effect, with an oviposition repellent rate of 46.43%.

### Toxicity of combined extracts from *A. belladonna* and *B. riparia* var. *megacephala* against *P. xylostella*

**FIGURE 4:**
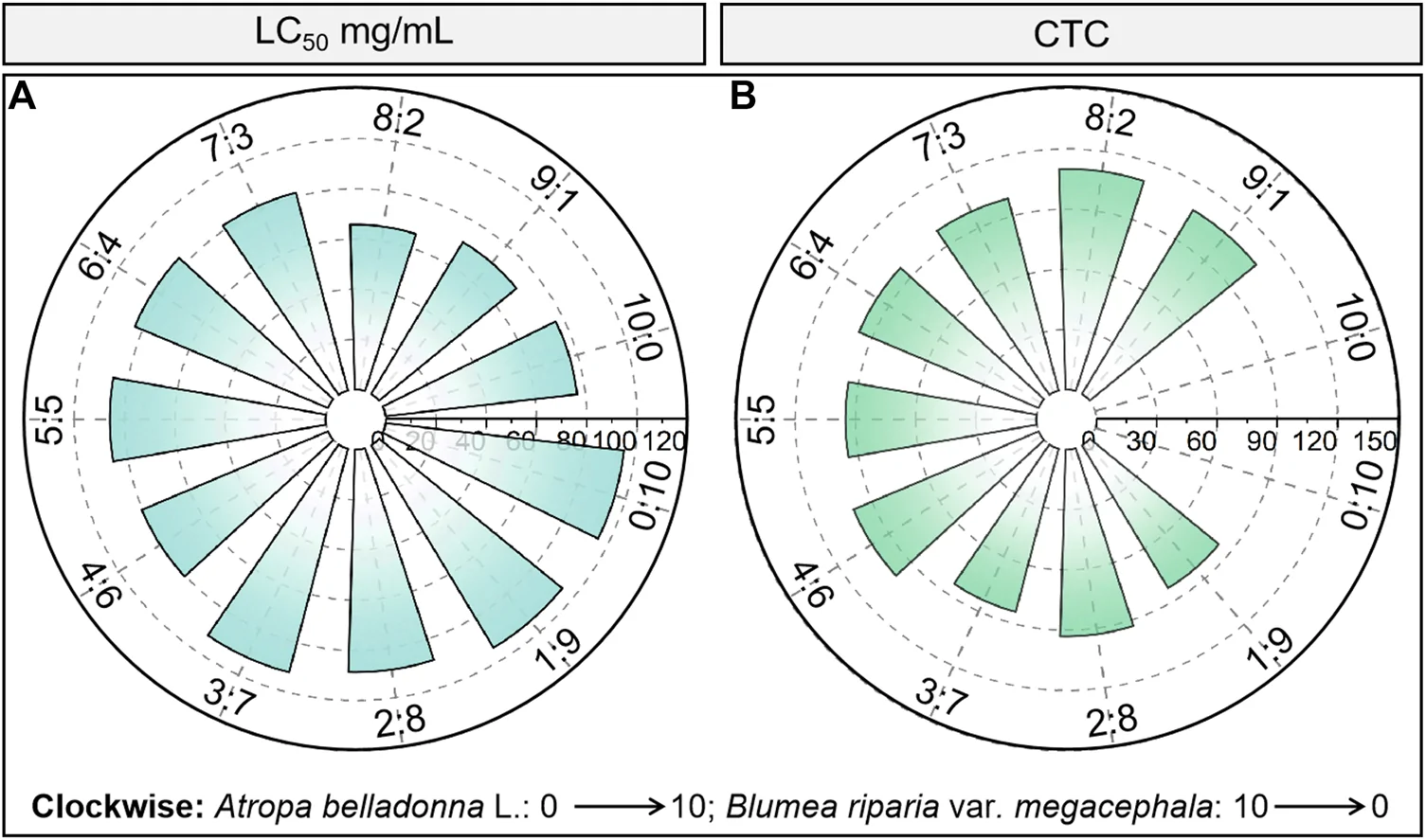
Toxicity of combined ethanol extracts of *A. belladonna* and *B. riparia* var. *megacephala* at different volume ratios against *P. xylostella* larvae after 72 h of exposure. **(A,B)** show the median lethal concentration (LC_50_) and co-toxicity coefficient (CTC), respectively. CTC values >120 indicate synergism, values between 80 and 120 indicate additive effects, and values <80 indicate antagonism.

Based on the above larvicidal, antifeedant, and oviposition-repellent bioassay results, *A. belladonna* and *B. riparia* var. *megacephala* exhibited the strongest overall activity among the 15 tested plant extracts. Therefore, these two extracts were selected for combination toxicity analysis, and a series of volume ratio gradients ranging from 10:0 to 0:10 was established to evaluate their toxicity and co-toxicity effects against third-instar P. xylostella larvae (Figures 4A,B). Probit analysis revealed that the LC50 values of *A. belladonna* alone (10:0) and *B. riparia* var. *megacephala* alone (0:10) were 76.69 mg/mL and 95.53 mg/mL, respectively, indicating greater intrinsic toxicity of the *A. belladonna* extract under the tested conditions.

The insecticidal effects of the combined extracts were ratio-dependent. Notably, the 8:2 (v/v) combination of *A. belladonna* to *B. riparia* var. *megacephala* yielded an LC50 of 65.76 mg/mL, lower than that of either single extract, indicating enhanced combined toxicity. The 9:1 combination also showed an LC50 of 70.67 mg/mL, outperforming both single extracts. In contrast, combinations with higher proportions of *B. riparia* var. *megacephala*, such as the 3:7 and 1:9 ratios, exhibited LC50 values of 92.16 mg/mL and 94.17 mg/mL, respectively, approaching the toxicity level of *B. riparia* var. *megacephala* alone.

As shown in Figure 4B, the CTC values further confirmed the ratio-dependent differences in interaction effects. The CTC values for the 8:2 and 9:1 ratios were 110 and 107, respectively, both within the additive range of 80–120 and near the upper boundary of this range. For the 7:3, 6:4, 5:5, and 4:6 ratios, CTC values ranged from 93 to 100, representing typical additive effects. The 3:7 and 1:9 ratios yielded CTC values of 84 and 83, respectively, only marginally above the antagonism threshold of 80. Importantly, none of the combined formulations exhibited CTC values below 80, confirming that no antagonistic interactions occurred.

### Formulation screening of EC prepared with *A. belladonna* and *B. riparia* var. *megacephala* extracts

Based on the laboratory toxicity and behavioral bioassay results, an 8:2 mixture of *A. belladonna* and *B. riparia* var. *megacephala* extracts was selected for the development of a botanical EC. The solvent type, surfactant type, and surfactant concentration were subsequently optimized for formulation preparation. As shown in Table 1, the combined extract was soluble in dimethyl sulfoxide, methanol, and ethanol, and the resulting solutions remained clear and stable under low-temperature conditions. In contrast, petroleum ether exhibited poor solubility and did not meet the homogeneity requirements for formulation development. Although dimethyl sulfoxide and methanol showed good solubilizing capacity, ethanol was selected as the optimal solvent because of its comparatively lower toxicity and favorable formulation stability.

**TABLE 1:** Screening test results of different solvents.

Sovlents	Solubility	Low temperature stability test	Quality qualification
Dimethyl sulfoxide	Soluble	Stable	Qualified
Methanol	Soluble	Stable	Qualified
Ethyl alcohol	Soluble	Stable	Qualified
Petroleum ether	Insoluble	—	Disqualified

During surfactant screening (Table 2), formulations containing pesticide emulsifier 500# and NP-2 remained homogeneous and transparent throughout the evaluation process, with no visible precipitation, phase separation, or oil separation observed. In contrast, formulations containing OP-10 exhibited slight precipitation under both low-temperature and thermal storage conditions, and precipitation became more pronounced after dilution, indicating reduced formulation stability. Emulsifier 601# also showed slight precipitation under low-temperature conditions, and its overall performance was inferior to that of emulsifier 500# and NP-2.

**TABLE 2:** Screening results of different surfactants.

Surfactants	Appearance	Low temperature stability	High temperature stability	Diluent stability
Pesticide emulsifier 500#	Homogeneous transparent liquid	Homogeneous transparent liquid	Homogeneous transparent liquid	Homogeneous transparent liquid
OP-10	Homogeneous non-transparent liquid	Microprecipitation	Little of precipitate	Precipitate
NP-2	Homogeneous transparent liquid	Homogeneous transparent liquid	Homogeneous transparent liquid	Homogeneous transparent liquid
Pesticide emulsifier601#	Homogeneous transparent liquid	Microprecipitation	Homogeneous transparent liquid	Homogeneous transparent liquid

Based on the preliminary screening results, concentration optimization experiments were conducted for the two selected emulsifiers, 500# and NP-2. As shown in Table 3, formulations containing emulsifier 500# at concentrations of 5%, 7.5%, and 10% remained homogeneous and transparent and satisfied the stability criteria under both thermal and low-temperature storage conditions. In contrast, formulations containing 2% or 2.5% emulsifier 500# exhibited turbidity, precipitation, and oil droplet separation, indicating reduced formulation stability and failure to meet the required standards.

**TABLE 3:** Pesticide emulsifier 500# effect of different content of surfactant.

Experimental conditions	Concentration				
	2%	2.5%	5%	7.5%	10%
Appearance	Turbid (disqualified)	Homogeneous transparent liquid (qualified)	Homogeneous transparent liquid (qualified)	Homogeneous transparent liquid (qualified)	Homogeneous transparent liquid (qualified)
Heat storage stability test	Amount of precipitate (disqualified)	Oil bead (disqualified)	Homogeneous transparent liquid (qualified)	Homogeneous transparent liquid (qualified)	Homogeneous transparent liquid(qualified)
Low temperature stability test	Turbid (disqualified)	Microprecipitation (disqualified)	Homogeneous transparent liquid (qualified)	Homogeneous transparent liquid (qualified)	Homogeneous transparent liquid (qualified)

The optimization results for NP-2 are presented in Table 4. Formulations containing NP-2 at concentrations of 8%, 16%, and 32% satisfied the stability requirements for EC formulations. However, formulations containing 4% NP-2 or lower exhibited turbidity and slight precipitation, indicating inadequate formulation stability. Considering both formulation performance and cost-effectiveness, the optimal concentrations were determined to be 5% for emulsifier 500# and 8% for NP-2.

**TABLE 4:** Stability test results of pesticide emulsifier NP-2 at different concentrations.

Experimental conditions	Concentration				
	2%	4%	8%	16%	32%
Appearance	White milky (disqualified)	Homogeneous transparent liquid (qualified)	Homogeneous transparent liquid (qualified)	Homogeneous transparent liquid (qualified)	Homogeneous transparent liquid (qualified)
Heat storage stability test	Oil bead (disqualified)	Homogeneous transparent liquid (qualified)	Homogeneous transparent liquid (qualified)	Homogeneous transparent liquid (qualified)	Homogeneous transparent liquid (qualified)
Low temperature stability test	Turbid (disqualified)	Microprecipitation (disqualified)	Homogeneous transparent liquid (qualified)	Homogeneous transparent liquid (qualified)	Homogeneous transparent liquid (qualified)

After systematic screening, the final EC formulation consisted of 10% active ingredients (8% *A. belladonna* extract and 2% *B. riparia* var. *megacephala* extract), 77% ethanol, 5% pesticide emulsifier 500#, and 8% NP-2. The EC formulation satisfied the physicochemical stability requirements for pesticide ECs under the tested conditions.

### Field efficacy of different treatments

**FIGURE 5:**
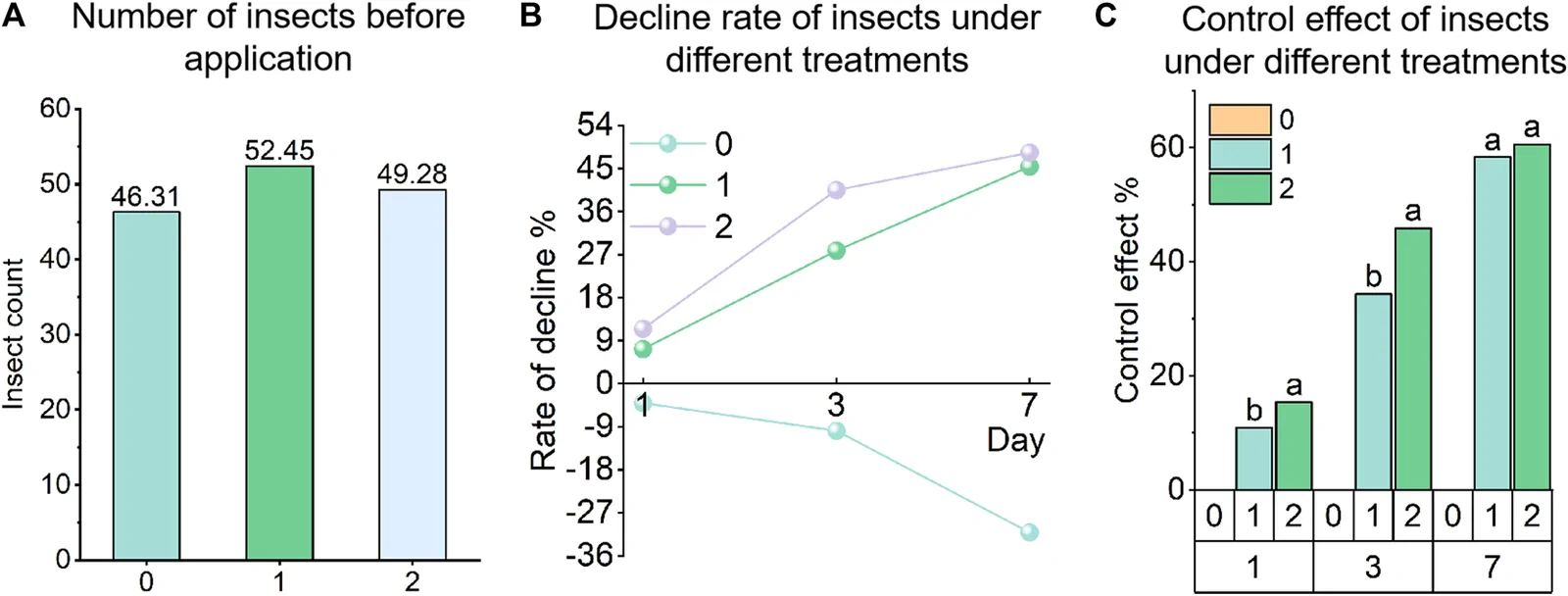
Field efficacy of different pesticide treatments against *P. xylostella* larvae. **(A)** shows larval population density before application, while **(B,C)** show larval population reduction rate and field control efficacy at 1, 3, and 7 days after application, respectively. Different letters indicate significant differences among treatments (independent-samples t-test, *p* < 0.05). Treatment 0: water spray (control); Treatment 1: 10% *A. belladonna-B. riparia var. megacephala* emulsifiable concentrate (EC); Treatment 2: 1% emamectin benzoate microemulsion.

Based on the formulation screening results described above, the 10% *A. belladonna-B. riparia* var. *megacephala* compound EC (Treatment 1) was evaluated for field control efficacy against *P. xylostella*. Pre-application surveys showed that the larval population density of *P. xylostella* in each treatment group ranged from 46.31 to 52.45 individuals per plot (Figure 5A), indicating relatively uniform pest density across the experimental field.

At 1 day post-application, clear differences in pest population reduction rates and control efficacy were observed among treatments (Figures 5B,C). Treatment 1 exhibited a pest population reduction rate of 7.24% and a field control efficacy of 10.86%. Treatment 2, the positive control emamectin benzoate, showed a reduction rate of 11.97% and a control efficacy of 15.41%. In contrast, the water-sprayed control group (Treatment 0) exhibited a population increase rate of 4.06%, indicating natural population growth. Statistical analysis demonstrated that the control efficacy of Treatment 2 was significantly higher than that of Treatment 1 at this time point (*P* < 0.05).

Over time, both the botanical compound EC and the chemical insecticide showed increasing trends in pest population reduction and control efficacy. At 7 days post-application (Figures 5B,C), Treatment 1 achieved a pest population reduction rate of 45.40% and a control efficacy of 58.35%, representing 5.3-fold and 4.5-fold increases, respectively, compared to day 1. Treatment 2 showed a reduction rate of 48.32% and a control efficacy of 60.58%, representing increases of 3.0% and 2.9%, respectively, compared to day 1. From day 1 to day 7, the increase in control efficacy was greater for Treatment 1 than for Treatment 2. Moreover, statistical analysis revealed that at day 7, there was no significant difference in control efficacy between Treatment 1 and Treatment 2 (Figure 5C, *P* > 0.05).

The water control group showed a population increase rate of 31.12%, with the *P. xylostella* population exhibiting marked growth in the absence of pesticide intervention, providing a strong contrast to the treated plots. In summary, the 10% *A. belladonna–B. riparia* var. *megacephala* compound EC exhibited a time-dependent field control pattern, with relatively low initial efficacy and improved later-stage efficacy under the tested field conditions.

## Discussion

The preliminary screening of 15 plant ethanol extracts showed clear interspecific differences in biological activity against *P. xylostella*. Among the tested species, *A. belladonna* and *B. riparia* var. *megacephala* consistently showed the strongest overall performance in larvicidal, antifeedant, and oviposition-repellent assays. The time-dependent increase in corrected mortality suggests that these extracts may provide sustained pest suppression and could serve as potential components of integrated pest management programs. For extracts such as *A. dauricus*, *A. ochotense*, *T. wattii*, and *E. japonicum*, the relatively small increase in mortality from 24 to 48 h may reflect lower acute toxicity or slower ingestion, ingestion, processing, and physiological response accumulation of their bioactive constituents in larvae, resulting in a more gradual insecticidal response rather than rapid mortality (Isman, 2006; Koul, 2008). However, the initial screening was conducted at a single concentration of 50 mg/mL; therefore, these results should be interpreted as a preliminary activity comparison rather than a complete concentration-response ranking among all tested plant species.

The relatively high insecticidal activity of *A. belladonna* may be partly associated with its reported tropane alkaloid profile. Previous phytochemical studies have shown that *A. belladonna* contains multiple alkaloids, including hyoscyamine, scopolamine, norhyoscyamine, and other tropane derivatives, while atropine and scopolamine have been shown to interact with insect muscarinic acetylcholine receptors (Hartmann et al., 1986). Therefore, these alkaloids may contribute to larval toxicity by interfering with cholinergic signaling and normal neural transmission in insects (Honda et al., 2007; Xia et al., 2016; Xie et al., 2025). The activity of *A. ochotense* may be partly associated with sulfur-containing compounds and steroidal saponins, which have been reported in *A. ochotense* and related *Allium* species (Go et al., 2024; Lee et al., 2018). These compound classes have been linked to oxidative stress and membrane permeation in insect systems (De Geyter et al., 2012; Zhang et al., 2017). The increase in activity observed for *B. riparia* var. *megacephala* over time may be associated with secondary metabolites, including volatile compounds previously reported in *Blumea* species. Previous studies have reported that essential oils from *Blumea* species may contain sesquiterpenoids and other volatile constituents with insecticidal properties (Qi et al., 2024). Similarly, the activity observed for *B. championi* may be associated with flavonoid compounds that have previously been reported to affect metabolic enzyme activity in insects (Gudavalli et al., 2024). These mechanistic explanations should be regarded as literature-supported hypotheses, because chemical profiling of the tested extracts was not performed in the present study.

The antifeedant and oviposition-repellent results further indicate that the two most active extracts may affect *P. xylostella* through multiple biological pathways. The sustained antifeedant activity of *A. belladonna* may be associated with tropane alkaloids that have previously been reported to interfere with neural signaling and feeding behavior in insects (Hartmann et al., 1986; Xia et al., 2016; Xie et al., 2025). Similarly, the activity of *B. riparia* var. *megacephala* may be discussed in relation to flavonoids and sesquiterpene-rich volatile constituents reported in *B. riparia* and *B. megacephala*. Chemical profiling studies have identified diverse flavonoids in *B. riparia* and *B. megacephala*, while GC/MS-FID analysis showed that sesquiterpene hydrocarbons and oxygenated sesquiterpenes are major chemical classes in *B. riparia* essential oils (Su et al., 2023; The et al., 2022). These compound classes have been associated with insecticidal or antifeedant activity through mechanisms such as feeding deterrence, digestive-enzyme inhibition, and detoxification-enzyme interference in insects (Muñoz-Núñez et al., 2024; Pereira et al., 2024). For other active extracts, the antifeedant activity of *A*. *ochotense*, *A*. *dauricus*, and *B*. *championi* may be discussed in relation to phytochemical groups reported from the corresponding species or genera, including sulfur-containing compounds and steroidal saponins in *A. ochotense* (Go et al., 2024), steroidal saponin-related constituents in *Asparagus* species (Güvenç, 1997), and flavonoids in *B. championi* (Chen et al., 1984). Specifically, sulfur-containing compounds in *A*. *ochotense* may interfere with pest gustatory perception by releasing irritant volatile substances, thereby affecting feeding behavior (Mann et al., 2011). Furthermore, flavonoids in *B*. *championi* may reduce nutrient utilization efficiency and indirectly inhibit feeding by affecting the activity of digestive enzymes in insects (Shalom et al., 2025).

The oviposition assay showed that *B. riparia* var. *megacephala* and *A. belladonna* not only affected larval survival and feeding behavior, but also reduced egg-laying by adult moths. Volatile metabolites previously reported in *B. riparia* species may partly explain this repellent effect (The et al., 2022). Meanwhile, beyond their possible neurotoxic effects on larvae, alkaloids reported in *A. belladonna* may interfere with oviposition site selection in lepidopteran adults through surface residues or chemical signals, thereby reducing oviposition preference. This interpretation is consistent with findings by Petzel-Witt et al. (2023) on the disruptive effects of plant alkaloids on adult oviposition behavior. Plant secondary metabolites such as flavonoids, alkaloids, and saponins have also been reported to reduce oviposition preference by affecting adult chemosensory processes or altering host plant volatile profiles (Zheng et al., 2023). Therefore, the strong performance of *A. belladonna* and *B. riparia* var. *megacephala* suggests multi-level pest control potential, including larvicidal activity, feeding inhibition, and oviposition repellency.

Combining botanicals can generate complementary or additive interactions among their bioactive components, thereby improving overall pest control efficacy and broadening the biological effects of botanical formulations (Rezaee Danesh et al., 2025). The combination toxicity assay showed that the biological effects of the two extracts were strongly influenced by their mixing ratio. The lower LC50 value of the 8:2 mixture compared with either single extract indicates enhanced combined toxicity. However, according to the CTC criteria, all tested combinations fell within the additive range rather than showing true synergism. Therefore, the interaction between *A. belladonna* and *B. riparia* var. *megacephala* should be interpreted as additive rather than synergistic. The relatively high CTC values of the 8:2 and 9:1 combinations suggest that a higher proportion of *A. belladonna* may be more conducive to eliciting complementary gains between the two components. In contrast, mixtures containing larger proportions of *B. riparia* var. *megacephala* showed LC50 values closer to that of *B. riparia* var. *megacephala* alone, suggesting that excessive reduction of the more toxic *A. belladonna* fraction weakened the overall lethal activity of the mixture. Importantly, no CTC value below 80 was observed, indicating that the two extracts were compatible for combination use and did not exhibit antagonistic interactions.

The ratio-dependent toxicity of the combined extracts, together with the larvicidal, antifeedant, and oviposition-repellent activities observed in the present study, suggests that the biological effects of *A. belladonna* and *B. riparia var. megacephala* are unlikely to be explained by a single response pathway alone. Instead, the two extracts may contain constituents that contribute to both lethal toxicity and behavioral interference, although the active compounds were not identified in this study. Recent compound-level studies on *P. xylostella* provide a useful mechanistic reference for this interpretation. For example, Singh et al. (2023) selected ethyl gallate and methyl gallate from reported *Triadica sebifera* leaf constituents as behaviorally active candidates and showed, through molecular docking, molecular dynamics, and steered molecular dynamics simulations, that these compounds could interact with olfaction-associated targets, including OBP1 and OR1. Their subsequent bioassays confirmed feeding-deterrent activity against *P. xylostella* larvae. These findings suggest that plant-derived compounds can potentially interfere with olfaction-related pathways in *P. xylostella*, which may partly explain how botanical extracts affect feeding or oviposition-related behaviors. However, because the active constituents of the present extracts were not identified, this explanation should be regarded as a possible mechanism that requires further validation. In addition, the NatProCP database reported by Mahanandia et al. (2026), which integrates plant-derived natural compounds with structural, ADMET, drug-likeness, and bioactivity information, illustrates how crude plant resources can be further translated into compound-level screening and target-based validation. Therefore, although the present work identifies promising crude extracts and an optimized botanical EC formulation, future studies should combine LC-MS or GC-MS profiling, bioassay-guided fractionation, and target-based validation to determine which constituents are responsible for the observed insecticidal, antifeedant, and oviposition-repellent activities. Such characterization will also be essential for evaluating residue behavior, non-target safety, and the practical development potential of this formulation.

The formulation screening results showed that ethanol, emulsifier 500#, and NP-2 were suitable components for preparing the botanical EC under the tested conditions. Ethanol was selected as the solvent because it provided good solubilizing capacity and comparatively lower toxicity than dimethyl sulfoxide and methanol. The final EC formulation, consisting of 10% active ingredients, 77% ethanol, 5% pesticide emulsifier 500#, and 8% NP-2, showed acceptable physicochemical stability in the high- and low-temperature tests. The use of ethanol and selected surfactants may help maintain the physical stability and biological activity of the botanical EC under the tested conditions. However, the stability tests conducted in this study provide only preliminary evidence of formulation stability and should not be regarded as a complete shelf-life evaluation. Further studies are needed to assess long-term storage stability, active-constituent degradation, emulsion stability after dilution, compatibility with field application practices, and performance under different environmental conditions.

The field trial provided preliminary evidence that the 10% *A. belladonna–B. riparia* var. *megacephala* compound EC could suppress *P. xylostella* populations under field conditions. The botanical EC showed weaker initial control efficacy than emamectin benzoate, which may be because emamectin benzoate acts on insect glutamate-gated chloride channels, interferes with neural signal transmission, and causes paralysis, thereby exhibiting relatively rapid insecticidal effects shortly after application (Wang et al., 2023). In contrast, the botanical compound EC may contain bioactive constituents such as alkaloids and sesquiterpenoids based on previous reports, which may contribute to delayed biological effects such as feeding inhibition, growth disturbance, and sustained toxicity. This may explain why the botanical EC showed weaker initial control efficacy than emamectin benzoate but continued to improve over time.

At 7 days post-application, the botanical EC reached a statistically comparable level to emamectin benzoate under the tested field conditions. Nevertheless, the absolute control efficacy of 58.35% should be interpreted as moderate field efficacy rather than strong field performance. The improved later-stage efficacy of the botanical formulation may be related to the gradual expression of toxic, antifeedant, and oviposition-repellent effects of the *A. belladonna* and *B. riparia* var. *megacephala* extracts, resulting in a time-dependent control pattern. This pattern differed from the faster initial response observed for emamectin benzoate under the tested conditions (Kaur et al., 2023). These results suggest that the botanical EC has promising but still improvable potential as a plant-derived control candidate for *P. xylostella* in cruciferous vegetable production. However, the observed control efficacy should be interpreted as preliminary field evidence rather than direct evidence of commercial viability.

This application potential should also be interpreted with caution. *A. belladonna* is known to contain tropane alkaloids, which may pose safety risks to humans, livestock, and non-target organisms at sufficient exposure levels. Therefore, natural origin should not be equated with inherent safety. Before practical agricultural application, further studies are needed to evaluate mammalian toxicity, phytotoxicity to crop plants, non-target organism effects, including beneficial insects, residue behavior, environmental fate, and ecological risks of this formulation. In addition, resistance development was not evaluated in the present study, and long-term selection experiments and field monitoring will be required to determine the role of this formulation in resistance management. Further optimization of formulation composition, application dosage, and application timing may help improve its field performance. Moreover, this field trial was conducted at a single experimental site and during one growing season. The observed field efficacy may therefore be influenced by local climatic conditions, pest population dynamics, and crop management practices. Multi-location and multi-season trials, together with optimization of formulation composition, application dosage, and application timing, will be required to verify the robustness, safety, and broader practical applicability of the botanical formulation.

## Conclusion

This study identified *A. belladonna* and *B. riparia var. megacephala* as the most promising botanical sources among the tested Qinling Mountain plant extracts for the management of *P. xylostella*. The two extracts showed complementary insecticidal, antifeedant, and oviposition-repellent activities, suggesting that their pest-control effects may involve multiple biological pathways. Their optimized combination further improved larval control without showing antagonistic interaction, supporting its suitability for botanical formulation development. The resulting botanical EC showed acceptable physicochemical stability and measurable field-control potential under the tested conditions. Overall, the findings of this study indicate that this botanical formulation has potential for further development in the control of *P. xylostella*. However, to achieve commercial application, it still needs to be validated through formulation optimization, long-term formulation stability tests, safety evaluation, and multi-site, multi-season field trials.

## Data Availability

The original contributions presented in the study are included in the article/supplementary material, further inquiries can be directed to the corresponding author.
